# Environmental and sociocultural factors are associated with pain-related brain structure among diverse individuals with chronic musculoskeletal pain

**DOI:** 10.21203/rs.3.rs-3425338/v1

**Published:** 2023-10-18

**Authors:** Lisa Domenico, Jared Tanner, Angela Mickle, Ellen Terry, Cynthia Garvan, Song Lai, Hrishikesh Deshpande, Roland Staud, David Redden, Catherine Price, Burel Goodin, Roger Fillingim, Kimberley Sibille

**Affiliations:** University of Florida; University of Florida; University of Florida; University of Florida; University of Florida; University of Florida; University of Alabama at Birmingham; University of Florida; University of Alabama at Birmingham; University of Florida; Washington University in St. Louis; University of Florida; University of Florida

**Keywords:** Environmental, Sociocultural, Ethnicity/Race, Musculoskeletal Pain, Neuroimaging

## Abstract

Chronic musculoskeletal pain is a leading cause of disability worldwide. Previous research indicates ethnic/race groups are disproportionately affected by chronic pain conditions. However, when considering socioenvironmental factors these disparities are no longer observed. Ethnic/race group differences have also been reported in pain-related brain structure. Given that environmental and sociocultural factors influence biology and health outcomes, this study aimed to investigate possible environmental and sociocultural contributions to structural differences in pain-related brain regions. A total of 147 non-Hispanic black and non-Hispanic white, middle and older aged adults with knee pain in the past month and a brain MRI are included in the analyses. Individuals also provided information specific to health and pain history and environmental and sociocultural resources. In hierarchical multiple regression models, sociocultural and environmental factors explained 6%−37% of the variance in thickness of *pain-related brain regions*, with seven of the eight brain regions being statistically significant. In the amygdala, hippocampus, insula, bilateral primary somatosensory cortex, and thalamus, ethnicity/race provided an additional 4%−13% of explanatory value. In the rostral/caudal anterior cingulate and dorsolateral prefrontal cortex, ethnicity/race was not a predictor after accounting for environmental, sociocultural, and other demographic measures. Findings inform health disparities research by elucidating the complexity of factors contributing to previously reported ethnicity/race group differences.

## 1. Introduction

Recent Global Burden of Disease data reports that over 1.71 billion people have chronic musculoskeletal (MSK) conditions worldwide, with osteoarthritis affecting over 343 million individuals [1]. Chronic MSK pain is a leading cause of physical disability and contributes to mental health decline, reduced work productivity, and loss of participation in life activities [2–4]. Despite increased focus from the scientific and healthcare communities and recent advancements in diagnostic imaging and therapeutic intervention, chronic MSK pain remains a significant impediment to health and well-being.

A significant amount of research indicates different ethnic/race groups are disproportionately affected by chronic pain conditions. Non-Hispanic Black (NHB) individuals consistently report higher pain severity [5–12], greater degree of disability [7, 13–21], and decreased sense of control over pain [14, 22, 23] compared to their non-Hispanic White (NHW) peers. Depressive symptoms are also associated with chronic pain conditions within NHB individuals [6, 15]. However, the relationship between ethnicity/race and pain is more complex. Imbalances in the representation by different ethnic/race groups from different sociocultural and environmental backgrounds have likely contributed to observed disparities [24]. We and others have shown that with consideration for socioenvironmental factors, previously reported ethnic/race group disparities wane [25].

Structural differences in brain regions involved in pain processing are associated with chronic pain. These differences have been observed in the rostral and caudal anterior cingulate cortices (rACC, cACC), amygdala, hippocampus, insula, medial prefrontal cortex (MPFC), dorsolateral prefrontal cortex (DLPFC), thalamus and the bilateral primary somatosensory cortex (S1) [26–30]. Ethnic/race differences in brain structure have been reported in the current study sample [31–33]. Identifying the contribution of environmental and sociocultural factors to observed pain-related brain structure differences is a critical step in addressing health disparities [2, 34].

Guided by the NIA and NIMHD Health Disparities Research Frameworks [2, 35], the current study aims to investigate environmental and sociocultural contributions to structural differences in pain-related brain regions among a diverse sample of individuals with knee pain consistent with or at risk for osteoarthritis. Regions of interest (ROIs) within the brain were selected based on findings from our previous work [29, 31, 32, 36] and the existing literature [26–28, 30]. Areas investigated include the rostral and caudal anterior cingulate cortices (ACC), insula, medial prefrontal cortex (MPFC), bilateral primary somatosensory cortex (S1), dorsolateral prefrontal cortex (DLPFC), thalamus, amygdala, and hippocampus. It is hypothesized that environmental and sociocultural factors will explain a statistically significant proportion of the variance of previously reported ethnic/race group differences in cortical thickness of ROIs.

## 2. Method

### 2.1. Study population

The study is a cross-sectional analysis of participants recruited for the Understanding Pain and Limitations in Osteoarthritic Disease-2 study (UPLOAD-2). Community-dwelling adults between ages 45–85 years old who self-identified as NHB or NHW and presented with unilateral or bilateral knee pain in the past month from the University of Florida (UF) and the University of Alabama at Birmingham (UAB) who completed brain imaging. Participants were recruited via multiple advertisement methods and clinic-based methods, as previously reported between August 2015 and May 2017 [37]. All procedures were reviewed and approved by the University of Florida Institution Review Board (IRB approval number-201400209) on June 6, 2014, and the University of Alabama at Birmingham Institution Review Board (IRB approval number-40915002) on November 11, 2014, and followed the guidelines of the Declaration of Helsinki. All participants provided verbal and written informed consent. Only participants who completed brain imaging were included in the analysis. This study follows the STROBE guidelines for reporting studies [38].

### 2.2. Procedures

Participants completed three study session visits which included a baseline health assessment, quantitative sensory testing, and brain imaging. All three sessions were completed with approximately one week between each session. Anthropometric measurements were obtained including waist circumference. Additionally, questionnaires acquired information on participants’ health history, pain history, disability, sociocultural factors, psychosocial factors, and risk factors. The measures described are limited to those relevant to the current study. Measures were selected that met criteria within the NIH/NIMHD Frameworks of environmental or sociocultural that were previously collected in the primary study.

### 2.2. Measures

#### 2.2.1. Clinical pain and disability

Total Pain Sites (*n* = 147). Participants were asked if they had pain on more days than not over the past three months at bilateral sites across the body (0–28 sites). Pain sites served as a covariate for global pain severity in the model since the other pain measures were limited to the knee [39]. Increasing number of pain sites has been linked to worse health outcomes and three or more pain sites are considered widespread pain [40, 41].

#### 2.3.2. Brain imaging

##### MRI Acquisition

Individuals who completed a brain MRI were included in this cross-sectional analysis. Both sites (UAB and UF) acquired MRI data using a 3 Tesla Philips Achieva scanner (32-channel head coil at UF and an 8-channel head coil at UAB). T1-weighted 3D three-dimensional magnetization-prepared rapid acquisition gradient-echo (MP-RAGE) images were acquired and used for analyses (TR: 7.0 ms, TE: 3.2 ms, flip angle: 8°, 1mm iso voxels, FOV: 240×240×176, sagittal acquisition).

##### MRI Processing

MP-RAGE files were processed using FreeSurfer 6.0 [42]. FreeSurfer is a set of software tools for the study of cortical and subcortical anatomy [43–45]. Segmentation of subcortical and related structures (including hippocampus, amygdala, and thalamus) was performed. The cerebral cortex was parcellated into units with respect to gyral and sulcal structure [46–48]. Procedures for the measurement of cortical thickness have been validated against histological analysis [49] and manual measurements [48, 50]. FreeSurfer morphometric procedures have been demonstrated to show good test-retest reliability across scanner manufacturers and across field strengths [51, 52]. MRI data were assessed for quality and participants were excluded for missing or insufficient quality data.

##### Brain Structure

Participants reported knee pain consistent with or at risk for knee osteoarthritis, and also reported pain in other body sites (Mean = 6, Range 0–28 additional sites). As such, analyses were guided based on previously identified brain areas in a systematic review for musculoskeletal pain[27] and other musculoskeletal and chronic pain research[26, 28, 30]. The final areas were selected a *priori* by a team consensus and align with our other work[29]. Mean thickness values for each cortical region (Desikan-Killiany-Tourville parcellation) and subcortical volumes were also exported to SPSS (IBM, Version 25) for analyses. Metrics were the bilateral mean thickness for the rostral and caudal anterior cingulate cortices (ACC), insula, medial prefrontal cortex (MPFC), primary somatosensory cortex (S1), dorsolateral prefrontal cortex (DLPFC), and thalamus, amygdala and hippocampus volumes adjusted for total intracranial volume.

#### 2.3.3. Environmental and Sociocultural measures

Environmental and sociocultural measures included self-reported educational level, current income, number of people living in the household, employment status, current insurance status, perceived social support and experiences of interpersonal discrimination.

##### Educational level (n = 147)–

Participants reported their attained level of education according to six categories: 1=“some school but did not complete high school,” 2=“high school degree,” 3=“two-year college degree,” 4=“four-year college degree,” 5=“master’s degree,”, and 6=“doctoral degree.”

##### Income level (n = 144)–

Annual household income was assessed in increments of $9,999, starting at 1= “$0–$9,999” and continuing until the last category: 10= “$150,000 or higher.”

##### Household size (n = 144)-

Participants were asked “Including you, how many people are living or staying at your home address?”.

##### Employment status (n = 147)–

Employment status was assessed using seven categories: “working now,” “only temporarily laid off, on sick leave or maternity leave”, “looking for work,” “unemployed”, “retired”, and “disabled, permanently or temporarily”, “student,” and “other.” Categories were then dichotomized into 1=“working now” or “retired” or 0=“only temporarily laid off, on sick leave or maternity leave”, “looking for work,” “unemployed”, “disabled, permanently or temporarily”, “student,” and “other”.

##### Insurance Status (n = 147)-

Participants were asked “Are you covered by health insurance or some other kind of health care plan?” as either 1 = yes or 0 = no. Participants who reported “unsure” were counted as missing.

##### The Multidimensional Scale of Perceived Social Support (MSPSS) [53] (n = 136)-

The MSPSS measures the perceived social support from family, friends and significant other using a 7-point Likert scale (1=“very strongly disagree” to 7=“very strongly agree”). Total scores are calculated as a summation of all questions with higher scores indicating greater perceived social support.

##### Experiences of Discrimination (EOD) questionnaire [54, 55] (n = 145)-

The EOD measures incidences of self-reported experiences of interpersonal discrimination over an individual’s lifetime, as well as the frequency of each event, worry for each event, the reason certain events occurred, and response to certain situations on a 0=“never”, 1=“once, 2.5=“2 to 3 times”, and 5=“4 or more times” scale. These values are summed with higher scores signifying greater experiences of interpersonal discrimination over an individual’s lifetime.

### 2.4. Statistical analysis

All data were analyzed using SAS v.9.4 (Cary, NC) and SPSS 26.0 (IBM, Chicago, IL), and checked for normality, outliers, and missing values. Differences between participant characteristics by sociodemographic (NHB and NHW) groups were analyzed using T-Test for continuous variables and Chi-Squared or Fisher Test where appropriate for categorical variables. A total of 147 participants completed brain imaging. Income (n = 3) and household number (n = 3) were imputed from data at a second timepoint. Individuals missing two or less questions on the perceived social support was imputed by using the within average of individual questions (n = 8). For individuals with more than 3 questions or more missing imputation of perceived social support (n = 2) and discrimination (n = 1) was imputed from data at a second timepoint. Two participants were missing data for perceived social support or discrimination and were excluded from analysis for a final sample size of n = 145. A sensitivity test repeating all analyses (n = 129) was completed excluding individuals with imputed data to confirm findings. Consistent with findings from our previous studies, primary explanatory variables in the model include: age, sex (1 = male, 2 = female), study site (1 = UF or 2 = UAB to account for possible scanner differences), waist circumference and total pain sites. Outcome measures for the brain ROIs: ACC, insula, MPFC, S1, DLPFC, thalamus, amygdala, and hippocampus. Nested Linear Regression Modeling was completed as follows: model 1) the primary explanatory variables, including age, sex, study site, waist circumference and total pain sites; model 2) primary explanatory variables from model 1 plus environmental and sociocultural variables including education, income, household number, employment, insurance status, social support and discrimination; model 3) all variables from model 2 plus sociodemographic groups, 1 = NHB adults with low sociodemographic resources and 2 = NHW adults with high sociodemographic resources.

## 3. Results

### 3.1. Participant characteristics.

Participant characteristics are displayed in Table 1. NHB adults were significantly younger with a greater number of socioenvironmental risk factors compared to the NHW adults. As each ethnic/race group has an incomplete representation, comparing by ethnic/race group classification may contribute to misinterpretations. As such, the term sociodemographic groups is used.

### 3.2. Associations between environmental and sociocultural factors and pain-related brain regions.

Nested Linear Regression models for ROIs are displayed in Table 2.

#### 3.2.1. Rostral and caudal anterior cingulate cortices (rACC and cACC) Thickness.

With all variables included, the overall model explained 17% of the variance in ACC thickness. Primary explanatory variables in model 1 accounted for 16% of the variance (F (5, 139) = 6.44, *p* < .0001). Environmental and sociocultural variables entered in model 2, explained < 1% of the variance in ACC thickness (*F* (12, 132) = 3.28, *p* = .0004). Sociodemographic groups entered in model 3 explained < 1% of the variance (*F* (13, 131) = 3.21, *p* = .0003). In the final model, age (*beta*=−.261, *p* = .0052), sex (*beta* = .178, *p* = .0309), and study site (*beta* = .164, *p* = .0419) remained significant. In a sensitivity analysis excluding those with *imputed* variables, findings were similar with the addition of lifetime discrimination (beta = .213, *p* = .023) indicated as significant while study site was no longer significant.

### 2.4 Insula.

With all variables included, the overall model explained 23% of the variance in insula thickness. Primary explanatory variables in model 1 accounted for 10% of the variance (*F* (5, 139) = 4.27, *p* = .0012). Environmental and sociocultural variables entered in model 2, explained 6% of the variance in insula thickness (*F* (12, 132) = 3.31, *p* = .0003). Sociodemographic groups entered in model 3 explained 7% of the variance (*F* (13, 131) = 4.33, *p* < .0001). In the final model, age (*beta*=−.289, *p* = .0013), study site (*beta* = .192, *p* = .0138), waist circumference (*beta*=−.239, p = .0019), lifetime discrimination (*beta* = .187, *p* = .0420), and sociodemographic groups (*beta* = .335, *p* = .0004) remained significant. In a sensitivity analysis excluding those with imputed variables, findings remained the same with the addition that perceived social support (*beta*=−.175, *p* = .040) also showed significance.

#### 3.2.5. Medial Prefrontal Cortex (MPFC) Thickness.

The overall models were not statistically significant (*p* = .1304, *p* = .3431, *p* = .2858). In a sensitivity analysis excluding those with imputed variables, none of the models were significant (*p* = .233, *p* = .454, *p* = .368).

#### 3.2.6. Bilateral Primary Somatosensory Cortex (S1) Thickness.

With all variables included, the overall model explained 24% of the variance in bilateral S1 thickness. Primary explanatory variables in model 1 accounted for 6% of the variance (*F* (5, 139) = 2.76, *p* = .0208). Environmental and sociocultural variables entered in model 2, explained 6% of the variance in bilateral S1 thickness (*F* (12, 132) = 2.56, *p* = .0045). Sociodemographic groups entered in model 3 explained 13% of the variance (*F* (13, 131) = 4.50, *p* < .0001). In the final model, age (*beta*=−.285, *p* = .0015), study site (*beta*=−.203, *p* = .0088), income (*beta* = .220, *p* = .0491) and sociodemographic groups (*beta* = .442, *p* < .0001) remained significant. In a sensitivity analysis excluding those with imputed variables, findings were similar however, income was no longer significant.

#### 3.2.7. Dorsolateral Prefrontal Cortex (DLPFC) Thickness.

Primary explanatory variables in model 1 accounted for 7% of the variance (*F* (5, 139) = 3.18, *p* = .0095). Model 2 (*F* (12, 132) = 1.83, *p* = .0487) and model 3 (*F* (13, 131) = 1.70, *p* = .0686) did not account for any additional variance. In a sensitivity analysis excluding those with imputed variables, no differences were identified.

#### 3.2.8. Thalamus Volume.

With all variables included, the overall model explained 39% of the variance in thalamus volume. Primary explanatory variables in model 1 accounted for 32% of the variance (*F* (5, 139) = 14.81, *p* < .0001). Environmental and sociocultural variables entered in model 2 explained 2% of the variance in thalamus volume (*F* (12, 132) = 7.24, *p* < .0001). Sociodemographic groups entered in model 3 explained 5% of the variance (*F* (13, 131) = 8.09, *p* < .0001). In the final model, age (*beta*=−.383, *p* < .0001), sex (*beta* = .342, *p* < .0001) and sociodemographic groups (*beta*=−.280, *p* = .0009) remained significant. In a sensitivity analysis excluding those with imputed variables, significant variables remained the same.

##### 3.2.9.2. Amygdala Volume.

With all variables included, the overall model explained 34% of the variance in amygdala volume. Primary explanatory variables in model 1 accounted for 30% of the variance (*F* (5, 139) = 13.16, *p* < .0001). Environmental and sociocultural variables entered in model 2, explained 1% of the variance in amygdala volume (*F* (12, 132) = 6.33, *p* < .0001). Sociodemographic groups entered in model 3, explained 3% of the variance (*F* (13, 131) = 6.80, *p* < .0001). In the final model, age (*beta*=−.330, *p* < .0001), sex (*beta* = .370, *p* < .0001), and sociodemographic groups (*beta* = − .246, p = .0049) remained significant. In a sensitivity analysis excluding those with imputed variables, significant variables remained with income (*beta* = .216, *p* = .041) also indicated.

##### 3.2.10.3. Hippocampus Volume.

With all variables included, the overall model explained 43% of the variance in hippocampus volume. Primary explanatory variables in model 1 accounted for 34% of the variance (F (5, 139) = 15.75, p < .0001). Environmental and sociocultural variables entered in model 2, explained 3% of the variance in hippocampus volume (*F* (12, 132) = 7.99, *p* < .0001). Sociodemographic groups entered in model 3, explained 6% of the variance (*F* (13, 131) = 9.31, *p* < .0001). In the final model, age (*beta*=−.236, *p* = .0024), sex (*beta* = .461, *p* < .0001), income (*beta* = .196, *p* = .0441), and sociodemographic groups (*beta*=−.311, *p* = .0002) remained significant. In a sensitivity analysis excluding those with imputed variables, significant variables remained with lifetime discrimination (*beta* = .174, *p* = .028) also showing significance.

## 4. Discussion

Guided by the NIA and NIMHD Health Disparities Research Frameworks [2, 35], the current study aimed to identify environmental and sociocultural contributions to previously reported ethnic/race group differences in pain-related brain structure in NHB and NHW adult reporting knee pain. As hypothesized, environmental and sociocultural factors were associated with cortical thickness in pain-related brain regions. The inclusion of sociodemographic groups (NHB and NHW) in the models frequently explained additional variance. Our results indicate that environmental and sociocultural factors contribute to some of the previously reported ethnic/race differences in cortical brain structure. The research approach aligns with the NIA and NIMHD Health Disparities Research Frameworks [35, 56] and identifies a combination of factors contributing toward previously described ethnic/race group differences in pain-related brain structure.

### 4.2. Environmental and sociocultural contributions to structural differences in brain regions associated with pain processing.

Associations between exposure to chronic pain and alterations in brain morphology are well-established [27, 57, 58]. Our previous publications in the same study sample indicated greater gray matter in the early stages of chronic MSK pain and lesser gray matter across cortical and subcortical areas of the brain with persisting, high stage chronic pain [31, 36]. Ethnicity/race was also identified as a significant factor with differences observed in brain morphology. The NHB individuals had thinner cortical thickness in multiple painrelated brain regions compared to their NHW peers [31]. In fact, a negative relationship was found between chronic pain severity and amygdala volume in the NHB group and a positive relationship was found within the NHW group. A similar pattern was shown in the same study sample related to pain catastrophizing [32]. Higher pain catastrophizing was associated with lower cortical thickness. Additionally, significant interactions were found between ethnicity/race, pain catastrophizing, and brain structure, with higher pain catastrophizing being associated with thinner S1 in NHW but not in NHB participants.

A key factor in both of these studies was that the NHB participants significantly differed on sociodemographic factors such as education, income, and healthcare access. The contributions of socioenvironmental factors in relation to health disparities are poorly quantified [2, 35, 56]. The purpose of the current investigation was by working with the same study sample, to incorporate available and recognized environmental and sociocultural variables and begin the process of systematically disentangling factors contributing to health related outcomes at the neurobiological level. As hypothesized, we show that after accounting for known confounding variables, environmental and sociocultural factors account for a significant proportion of the variability observed in pain-related brain structure, 0 to 6%, while the sociodemographically differing ethnic/race groups made a more limited contribution in five of the seven models with ranges from < 1 to 13%.

Age was the strongest and most consistent predictor in all of the clinical pain and pain-related brain structure models. Age-related changes in clinical pain and brain structure are well recognized. Less often considered is how the cumulative impact of environmental and sociocultural risk exposures might accelerate age-related brain changes also influencing clinical pain experiences. As environmental and sociocultural variables experienced over the lifespan influence brain structure, understanding and addressing health disparities will require that we improve and broaden the measures available to better capture environmental and sociocultural life experiences across the lifespan [59, 60].

### 4.3. Strengths, limitations, and future directions.

Study findings should be viewed in light of several strengths and limitations. The study benefitted from a large and ethnically diverse sample, with data collected from two study sites (Gainesville, Florida and Birmingham, Alabama). However, our findings are limited by a cross-sectional design. Longitudinal data would improve understanding of the relationships between environmental and sociocultural factors, and pain-related brain structure. Further, participants in the study had knee pain with or at risk for knee OA. As such, most of the participants reported mild to moderate chronic musculoskeletal pain.

Additionally, a number of the measures capturing environmental and sociocultural variables are categorical in nature and may not best capture the constructs of interest. Despite limitations in study design and measures, these findings lend strong support for a better understanding of the role that environmental and sociocultural factors play in the experience of chronic MSK pain and their biological consequences.

Future work would benefit from using additional comprehensive measures specifically designed to assess environmental and sociocultural factors, including measures that capture the cumulative exposure to environmental and sociocultural factors across the lifespan. Early as well as long-term exposure to detrimental environmental and sociocultural factors are likely to be more influential on neuroplasticity than exposures limited to adulthood. Additionally, as we continue to unravel factors contributing to health disparities and investigate possible ethnic/race group differences, confirming groups are similar based on socioenvironmental factors will be essential. When groups differ on relevant sociodemographic variables, describing findings as “ethnic/race group differences” contributes to misinterpretations and reduces the ability to identify and address the factors contributing to health disparities [31].

## 5. Conclusions

Ethnic/race group differences in pain-related experiences were well-established. However, a growing body of evidence indicates that when socioenvironmental factors are accounted for and groups are balanced across sociodemographic variables, previously observed ethnic/race differences are no longer indicated. Ethnic/race group differences have also been reported in pain-related brain structure. The contributions of environmental and sociocultural factors have been minimally investigated. Our findings show that by including a combination of environmental and sociocultural factors into our research models, previously reported ethnic/race group differences are reduced or not identified. Importantly, this work will help inform health disparities research by elucidating factors contributing to previously reported ethnicity/race group differences in pain-related brain structure.

## Figures and Tables

**Table 1:** Sample characteristics

Characteristics	Total	Non-Hispanic Black	Non-Hispanic White	p-value
	N = 145	N = 71	N = 74	
Primary Explanatory Variables				
Age, mean ± std	58.3 ± 8.0	56.2 ± 6.3	60.2 ± 9.0	**0.0020**
Sex, n (%)				0.2204
Male	52 (35.9)	29 (40.9)	23 (30.7)	
Female	93 (64.1)	42 (59.1)	52 (69.3)	
Waist Circumference, mean ± std	102.4 ± 14.3	102.2 ± 13.4	102.6 ± 15.2	0.8576
Study Site, n (%)				0.2930
University of Florida	93 (63.3)	42 (59.2)	50 (67.6)	
University of Alabama at Birmingham	54 (36.7)	29 (40.8)	24 (32.4)	
Number of Pain Sites, median [std]	6.0 [4.0]	5.8 [3.7]	5.5 [3.6]	0.5989
Environmental and Sociocultural Factors				
Annual household income, n (%)				< .0001
$0 - $9,999	34 (23.5)	24 (33.8)	10 (13.5)	
$10,000 - $19,999	17 (11.7)	11 (15.5)	6 (8.1)	
$20,000 - $29,999	20 (13.8)	12 (16.9)	8 (10.8)	
$30,000 - $39,999	6 (4.1)	4 (5.7)	2 (2.7)	
$40,000 - $49,999	12 (8.3)	3 (4.3)	9 (12.2)	
$50,000 - $59,999	16 (11.0)	5 (7.0)	11 (14.8)	
$60,000 - $79,999	13 (9.0)	4 (5.6)	9 (12.2)	
$80,000 - $99,999	11 (7.6)	5 (7.0)	6 (8.1)	
$100,000 - $149,999	11 (7.6)	2 (2.8)	9 (12.2)	
$150,000 or higher	5 (3.4)	1 (1.4)	4 (5.4)	
Education, n (%)				< .0001
Some school but did not complete high school	10 (6.9)	8 (11.3)	2 (2.7)	
High school degree	55 (38.0)	32 (45.1)	23 (31.1)	
Two-year college degree	25 (17.2)	12 (16.9)	13 (17.5)	
Four-year college degree	30 (20.7)	10 (14.1)	20 (27.0)	
Master’s degree	18 (12.4)	7 (9.8)	11 (14.9)	
Doctoral degree	7 (4.8)	2 (2.8)	5 (6.8)	
Employment status, n (%)				0.1056
Currently working/retired	100 (69.0)	44 (62.0)	56 (75.7)	
Laid off/on leave/ unemployed; looking/disabled/ student	45 (31.0)	27 (38.0)	18 (24.3)	
Number in household, mean ± std	2.2 ± 1.2	2.3 ± 1.4	2.1 ± 1.0	0.4312
Current Insurance, n (%)	125 (86.2)	60 (84.5)	65 (87.8)	0.6340
Multidimensional Scale of Perceived Social Support	65.3 ± 17.6	64.5 ± 19.6	66.0 ± 15.6	0.6107
Lifetime Discrimination	7.7 ± 9.4	12.5 ± 9.3	3.1 ± 7.0	< .0001

Between group differences were established using Independent Samples T-Test (two-tailed), Chi-Sq or Fisher’s Exact Test where appropriate

**Table 2:** Nested linear regression analyses examining environmental and sociocultural factors in relation to brain regions associated with pain Processing.

	ACC Thickness	Insula Thickness	MPFC Thickness	S1 Thickness	DLPFC Thickness	Thalamus Volume	Amygdala Volume	Hippocampus Volume
Independent Variables								
Model 1: Primary								
Age	−0.3402***	−0.2072*	−0.1933	−0.1851*	−0.2469**	−0.4359***	−0.3445***	−0.3009***
Sex	0.1787*	0.1319	0.0522	0.1004	0.0766	0.3347***	0.3830***	0.4464***
Study Site	0.1468	0.1700*	0.0710	−0.2334**	0.0099	−0.0153	0.1104	0.0532
Waist Circumference	−0.1125	−0.2287**	−0.0402	0.0041	−0.2057*	0.0158	−0.0241	0.0684
Total Pain Sites	−0.0325	−0.0020	−0.1412	−0.0745	−0.0590	0.0478	0.0460	0.0955
*Adj. R^2^*	0.1589	0.1020	-	0.0576	0.0703	0.3240	0.2969	0.3388
Model 2: Env/Soc								
Age	−0.2435**	−0.2459**	−0.1631	−0.2272*	−0.2526**	−0.4191***	−0.3618***	−0.2761***
Sex	0.1869*	0.0794	0.0380	0.0379	0.0502	0.3235***	0.3534***	0.4406***
Study Site	0.1608*	0.1829*	0.0757	−0.2152*	−0.0056	−0.0300	0.0897	0.0326
Waist Circumference	−0.0983	−0.2309**	−0.0267	−0.0005	−0.2012*	0.0339	−0.0095	0.0802
Total Pain Sites	−0.0030	0.0606	−0.1068	−0.0106	−0.0453	0.0615	0.0401	0.0917
Education	−0.0563	0.1109	0.0151	−0.0340	−0.0538	−0.0582	−0.1097	−0.1105
Income	0.1431	0.2320*	0.1472	0.3417**	0.2580*	0.0446	0.1208	0.1103
Household Size	0.1284	0.0712	0.0980	−0.0053	−0.0471	−0.0647	−0.0696	−0.0991
Employment	−0.0837	0.0019	−0.1461	−0.0227	−0.0698	0.1376	0.1239	0.1239
Insurance	−0.0955	0.0048	0.0112	−0.1251	−0.0102	−0.0687	0.0373	−0.079
Perceived Social Support	−0.0234	−0.1588	0.0572	−0.0287	−0.0300	0.0852	0.0079	0.0001
Discrimination	0.1100	0.0288	0.0244	−0.1173	0.1082	0.1911*	0.1822*	0.2325**
*Adj. R^2^*	0.1598	0.1616	-	0.1147	0.0650	0.3419	0.3077	0.3679
*ΔR^2^*	0.0009	0.0596	-	0.0571	−0.0053	0.0179	0.0108	0.0291
Model 3: Sociodemographic Group								
Age	−0.2614**	−0.2894**	−0.1823	−0.2845**	−0.2582	−0.3827***	−0.3299***	−0.2358**
Sex	0.1777*	0.0569	0.0281	0.0082	0.0473	0.3423***	0.3699***	0.4615***
Study Site	0.1645*	0.1920*	0.0797	−0.2033**	−0.0044	−0.0376	0.0830	0.0242
Waist Circumference	−0.1016	−0.2390**	−0.0303	−0.0112	−0.2023	0.0407	−0.0036	0.0877
Total Pain Sites	−0.0096	0.0445	−0.1139	−0.0318	−0.0474	0.0749	0.0519	0.1066
Education	−0.0772	0.0600	−0.0073	−0.1012	−0.0604	−0.0155	−0.0723	−0.0633
Income	0.1054	0.1400	0.1067	0.2205*	0.2459	0.1217	0.1883	0.1957*
Household Size	0.1335	0.0838	0.1035	0.0113	−0.0455	−0.0752	−0.0789	−0.1108
Employment	−0.0639	0.0502	−0.1248	0.0411	−0.0635	0.0971	0.0884	0.0790
Insurance	−0.0769	0.0501	0.0311	−0.0655	−0.0042	−0.1066	0.0041	−0.1209
Perceived Social Support	−0.0192	−0.1485	0.0617	−0.0151	−0.0287	0.0766	0.0004	−0.0094
Discrimination	0.1747	0.1868*	0.0941	0.0909	0.1289	0.0587	0.0663	0.0859
Sociodemographic Group	0.1373	0.3352***	0.1478	0.4416***	0.0438	−0.2808***	−0.2460**	−0.3110***
*Adj. R^2^*	0.1662	0.2313	-	0.2401	-	0.3903	0.3435	0.4287
*ΔR^2^*	0.0064	0.0697	-	0.1254	-	0.0484	0.0358	0.0608

Standardized (beta) values reported. n = 145. ACC = anterior cingulate cortices; MPFC = medial prefrontal cortex; S1 = thalamus and the bilateral primary somatosensory cortex; DLPFC = dorsolateral prefrontal cortex

* *p* < .05,

** *p* < .01,

*** *p* < .001

Note: Models 1–3 were not significant for MPFC, and Models 3 were not significant for DLPFC.

## Data Availability

Data is not publicly available but can be requested from the corresponding author.
